# Antinociceptive activity of petroleum ether fraction obtained from methanolic extract of *Clinacanthus nutans* leaves involves the activation of opioid receptors and NO-mediated/cGMP-independent pathway

**DOI:** 10.1186/s12906-019-2486-8

**Published:** 2019-04-02

**Authors:** Zainul Amiruddin Zakaria, Mohammad Hafiz Abdul Rahim, Mohd Hijaz Mohd Sani, Maizatul Hasyima Omar, Siew Mooi Ching, Arifah Abdul Kadir, Qamar Uddin Ahmed

**Affiliations:** 1 Department of Biomedical Science, Faculty of Medicine and Health Sciences, Universiti Putra Malaysia, 43400 Serdang, Selangor Malaysia; 2 Integrative Pharmacogenomics Institute (iPROMISE), Level 7, FF3, Faculty of Pharmacy, Universiti Teknologi MARA (UiTM), Puncak Alam Campus, 42300 Bandar Puncak Alam, Selangor Malaysia; 3 Phytochemistry Unit, Herbal Medicine Research Centre, Institute for Medical Research, 50588 Jalan Pahang, Kuala Lumpur Malaysia; 4 Department of Family Medicine, Faculty of Medicine and Health Sciences, Universiti Putra Malaysia, 43400 Serdang, Selangor Malaysia; 5 Department of Veterinary Pre–Clinical Science, Faculty of Veterinary Sciences, Universiti Putra Malaysia, 43400 Serdang, Selangor Malaysia; 6 Department of Pharmaceutical Chemistry, Faculty of Pharmacy, International Islamic University Malaysia, Jalan Istana, Bandar Indera Mahkota, 25200 Kuantan, Pahang Malaysia

**Keywords:** *Clinacanthus nutans*; Acanthaceae, Antinociceptive, Mechanisms of action, Opioid receptors, NO-mediated/cGMP-independent pathway; phenolic compounds

## Abstract

**Background:**

Methanol extract (MECN) of *Clinacanthus nutans* Lindau leaves (family Acanthaceae) demonstrated peripherally and centrally mediated antinociceptive activity via the modulation of opioid/NO-mediated, but cGMP-independent pathway. In the present study, MECN was sequentially partitioned to obtain petroleum ether extract of *C. nutans* (PECN), which was subjected to antinociceptive study with aims of establishing its antinociceptive potential and determining the role of opioid receptors and l–arginine**/**nitric oxide**/**cyclic-guanosine monophosphate (l–arg**/**NO**/**cGMP) pathway in the observed antinociceptive activity.

**Methods:**

The antinociceptive potential of orally administered PECN (100, 250, 500 mg/kg) was studied using the abdominal constriction-, hot plate- and formalin-induced paw licking-test in mice (*n* = 6). The effect of PECN on locomotor activity was also evaluated using the rota rod assay. The role of opioid receptors was determined by pre-challenging 500 mg/kg PECN (p.o.) with antagonist of opioid receptor subtypes, namely β–funaltrexamine (β–FNA; 10 mg/kg; a μ-opioid antagonist), naltrindole (NALT; 1 mg/kg; a δ-opioid antagonist) or nor–binaltorphimine (nor–BNI; 1 mg/kg; a κ-opioid antagonist) followed by subjection to the abdominal constriction test. In addition, the role of l–arg**/**NO**/**cGMP pathway was determined by prechallenging 500 mg/kg PECN (p.o.) with l–arg (20 mg/kg; a NO precursor), 1H-[1, 2, 4] oxadiazolo [4,3-a]quinoxalin-1-one (ODQ; 2 mg/kg; a specific soluble guanylyl cyclase inhibitor), or the combinations thereof (l–arg + ODQ) for 5 mins before subjection to the abdominal constriction test. PECN was also subjected to phytoconstituents analyses.

**Results:**

PECN significantly (*p* < 0.05) inhibited nociceptive effect in all models in a dose-dependent manner. The highest dose of PECN (500 mg/kg) also did not significantly (*p* > 0.05) affect the locomotor activity of treated mice. The antinociceptive activity of PECN was significantly (*p* < 0.05) inhibited by all antagonists of μ-, δ-, and κ-opioid receptors. In addition, the antinociceptive activity of PECN was significantly (*p* < 0.05) reversed by l–arg, but insignificantly (*p* > 0.05) affected by ODQ. HPLC analysis revealed the presence of at least cinnamic acid in PECN.

**Conclusion:**

PECN exerted antinocicpetive activity at peripheral and central levels possibly via the activation of non-selective opioid receptors and modulation of the NO-mediated/cGMP-independent pathway partly via the synergistic action of phenolic compounds.

## Introduction

Pain is an essential sensation that plays a vital role as a body’s natural defence system by alerting us to possible tissue injury while nociception is described as “the neural processes of encoding and processing noxious stimuli” that usually leads to pain [1]. The process mentioned above is initiated by specialized peripheral sensory neurons (nociceptors) that are activated by noxious stimuli (i.e., mechanical, thermal, and chemical stimuli) due to tissue injury and damage, and these nociceptors are usually found in the cutaneous tissues, bone, muscle, connective tissues, vessels and viscera. These stimuli are transduced into electrical impulses (action potentials) that are transmitted predominantly through Aδ- and C-fibre nociceptors (primary afferent neurons) into the dorsal horn of the spinal cord [1]. A variety of excitatory neurotransmitters are released by the primary afferent neurons, such as excitatory amino acids, protons, peptides, lipids and cytokines, and others, which act on their specific receptors and ion channels, to activate the second order neurons of the spinal dorsal horn. Once activated, the action potentials are then ascended to the thalamus and cerebral cortex through spinothalamic or other tracts that lead to perception of pain [2].

Pain can be modulated by various analgesic drugs that suppress pain signals by acting in various mechanisms on the peripheral (PNS) and central nervous system (CNS) [2]. However, various adverse effects have been reported with these agents. For instance, nonsteroidal anti-inflammatory drugs (NSAIDs) may cause gastrointestinal irritation and/or bleeding, decreased platelet aggregation (leads to prolong bleeding time), kidney damage, edema, bone marrow suppression, rashes, as well as anorexia. Opioids, on the other hand, may also lead to constipation, dizziness, nausea, respiratory depression, sedation, vomiting, as well as physical dependence and tolerance, with the most being constipation and nausea. Hence, there is an imperative need to discover new therapies that are more effective and safe with lesser or no side effects. For instance, natural product-based medications, particularly plant-derived, are believed to be a valuable source of chemical substances that promise to have a good potential therapeutic applicability [3].

*Clinacanthus nutans* (*C. nutans*) Lindau, known to the Malay as “*Belalai Gajah*”, is one of the medicinal plants that have received considerable attention from many research groups within the past few years. This small shrub belongs to the Acanthaceae family and commonly grows in tropical Southeast Asian countries. Local communities in the Southeast Asian countries including Malaysia have traditionally used *C. nutans* for the treatment of various disorders (e.g., burns, diabetes, diarrhea, fever, rheumatoid arthritis scalds etc.), including pain relief [4, 5]. Besides that, based on some recently published reviews *C. nutans* has also been scientifically proven to possess various pharmacological activities (i.e. antibacterial, anticholinesterase, anti–dengue, antidiabetes, antiherpes, anti–inflammatory, antimutagenic, antioxidant, antiproliferative, antitumor, anti–varicella–zoster and cytotoxic activities). In addition, a variety of phytochemical constituents have been detected in the leaves of *C. nutans*, such as flavonoids, phenolic acids, diterpenes, phytosterols, saponins, steroids, chlorophyll and its various derivatives [4, 5]. Previous study has reported on the antinociceptive activity and possible mechanisms of action of methanolic extract of *C. nutans* (MECN) [6, 7]. MECN was earlier demonstrated to exert antinociceptive activity at peripheral and central levels when assessed using the acetic acid-induced abdominal constriction-, hot plate- and formalin-induced paw licking-test. The antinociceptive activity was found to involve the activation of opioid receptors and was modulated via the NO-mediated, but cGMP-independent, systems when assessed using the abdominal constriction test [6]. Further investigation on the possible mechanisms of antinociceptive activity modulated by MECN using the abdominal constriction test revealed the non-selective action of the extract towards opioid receptors as indicated by the ability of μ-, δ-, and κ-opioid receptors antagonists to inhibit the action of MECN [7]. The extract was also found to exert its antinociceptive activity via the modulation of several non-opioid receptor systems namely dopaminergic,adenosinergic, cholinergic, α_2_-noradrenergic, or β-adrenergic receptors, and through the activation of different types of K^+^ channels namely voltage-activated-, Ca^2+^-activated, or ATP-sensitive-K^+^ channels based on the ability of their respective antagonist or blocker to inhibit the antinociceptive activity of MECN [7]. In addition, MECN was also found to inhibit capsaicin-, glutamate-, phorbol 12-myristate 13-acetate-, bradykinin-induced nociceptive response indicating the extract ability to modulate nociceptive transmission via the inhibition of protein kinase C-, bradykinin-, TRVP1 receptors-, and glutamatergic-signaling pathways. Being a solvent of intermediate polarity, methanol was able to extract both the polar and non-polar, as well as intermediate polar bioactive compounds, thus, make it impossible to predict on the nature of bioactive compounds responsible for the observed activity. Due to this matter and in an attempt to further establish the pain-relieving potential of *C. nutans*, MECN was further partitioned using petroleum ether, ethyl acetate and water to obtain a non-polar (PECN), intermediate polar (EACN) and polar (AQCN) partitions, respectively. Each of these partitions were then subjected to the same animal nociceptive model (abdominal constriction test) to determine their effectiveness and the most effective partition were then subjected to the other nociceptive models (hot plate test and formalin-induced paw licking test). Data from the preliminary screening revealed that the petroleum ether fraction (PECN) demonstrated the most effective antinociceptive activity in comparison to the other fractions of MECN (data not shown), which justifies its selection for further investigation on the pain-relieving potential of *C. nutans*. Therefore, the present study was carried out to establish the antinociceptive profile of petroleum ether fraction of *C. nutans* (PECN) and to determine the possible mechanisms of antinociception modulated by PECN using various nociceptive models.

## Methods

### Plant collection

The leaves of *C. nutans* were acquired from Clinnthus Enterprise (Kuala Lumpur, Malaysia) in January 2013 and authenticated by a certified botanist (Dr. Shamsul Khamis) from the Institute of Bioscience (IBS), Universiti Putra Malaysia (UPM), Serdang, Selangor, Malaysia. A voucher specimen (No. SK 2679/15) was deposited in the herbarium of IBS, UPM.

### Preparation of PECN

The preparation of methanolic extract has already been described in detail in our previous published study [6]. Meanwhile, the preparation of semi–purified petroleum ether fraction was done by using the procedure described by Zakaria et al. [8]. About 20 g of the dried methanolic extract was suspended in 1000 mL of methanol (MeOH; Fisher Scientific, UK), and then 200 mL of distilled water was added to afford an aqueous MeOH solution. The aqueous MeOH solution was further transferred into a 2000 mL separatory funnel (12 O.D. × 75 mm stem) and 700 mL of petroleum ether (PE; Fisher Scientific, UK) was added to the solution. The mixture solution was vigorously shaken thoroughly and then allowed to separate into two immiscible layers for 24 h. The supernatant of PE partition (lower layer) was collected and filtered by using Whatman No. 1 filter paper. The residue (aqueous MeOH solution) underwent the same procedure for three times until the supernatant of PE partition became colorless. Each supernatant of PE partition was then pooled together and evaporated by using a vacuum rotary evaporator (Hei–VAP Value; Heidolph, Schwabach, Germany) at 40 °C under reduced pressure, in order to obtain a concentrated and afforded extract of dried petroleum ether (PECN). The dried PECN was then stored at 4 °C until it was further used.

### Experimental animals

The animal experimentations were performed using the adult male ICR mice weighing between 25 and 30 g. The animals were cared, handled and provided housing facilities according to the detailed procedures described by Abdul Rahim et al. [6]. Briefly, the animals were obtained from the Animal Source Unit, Faculty of Veterinary Medicine, UPM, Serdang, Malaysia and kept in groups of six *per cage* in the Animal Holding Unit, Faculty of Medicine and Health Science, UPM, under standard environmental condition of 27 ± 2 °C, 70–80% humidity and 12 h light/dark cycle for at least 48 h prior to the experiment. The animals were allowed accessed to commercial food pellets (Gold Coin Feed Mills, Port Klang, Malaysia) and water ad libitum. The animal experimentations have been approved by the Institutional Animal Care and Use Committee (IACUC), UPM (Ref. Number UPM/IACUC/AUP–R032/2013) and were performed as per the animals ethical guidelines adopted from Zimmermann [9]. All efforts were made to minimize the animal suffering by applying the minimum intensities of noxious stimuli required to cause constant effects of the treatments. Experiments were conducted between 09:30 and 18:30 h to minimize the effects of environmental changes. At the end of the experiments, the animals were anesthetized with 60 mg/kg ketamine plus 7.5 mg/kg xylazine and euthanized by cervical dislocation.

### Drugs and chemicals

The following drugs, namely acetylsalicylic acid (ASA), morphine hydrochloride (MOR), diazepam (DZP), l–arginine and 1H-[1, 2, 4] oxadiazole [4,3–a]quinoxaline–1–one (ODQ) were purchased from Sigma–Aldrich (St. Louis, MO, USA), while β–funaltrexamine hydrochloride (β–FNA), nor–binaltorphimine dihydrochloride (nor–BNI), and naltrindole hydrochloride (NALT) were purchased from Tocris Bioscience (Ellisville, Missouri, USA). Acetic acid and dimethyl sulfoxide (DMSO) were purchased from Fisher Scientific (Fair Lawn, NJ, USA). Formaldehyde was obtained from R & M Chemicals (Essex, England). All drugs were dissolved in physiological saline (0.9% [*w*/*v*] NaCl) except for PECN, ASA, DZP and MOR, which were dissolved in 10% DMSO (*v*/*v*). The vehicle that was used alone had no effects per se on the nociceptive responses in mice. The other solutions (i.e., 0.6% acetic acid and 5% formalin) were prepared in 0.9% NaCl. All drugs, chemicals, PECN solutions were administered in 10 mL/kg volumes and were freshly prepared immediately prior to use.

### Evaluation of PECN effect on different models of nociception

#### Acetic acid–induced abdominal constriction test

The animals (*n* = 6) were treated with vehicle (10% DMSO, 10 mL/kg, per os (p.o.), negative control), ASA (100 mg/kg, p.o., positive control), or PECN (100, 250 or 500 mg/kg, p.o.) 60 min before the administration of phlogistic agent (0.6% acetic acid, 10 mL/kg, intraperitoneal (i.p.). The number of abdominal constrictions produced was counted from 5 min after acetic acid administration cumulatively for 25 min, as described previously in detail [10].

#### Hot plate test

In order to evaluate the central antinociceptive effect of PECN, a hot plate test was carried out according to the method previously described in detail by Sani et al. [10]. The animals (*n* = 6) were treated with vehicle (10% DMSO, 10 mL/kg, p.o.), MOR (5 mg/kg, p.o., positive control) or PECN (100, 250 or 500 mg/kg, p.o.) 60 min before the test.

#### Formalin–induced paw licking test

The procedure used was similar to the one used in our previous work [10]. Mice (*n* = 6) received vehicle (10% DMSO, 10 mL/kg, p.o.), ASA (100 mg/kg, p.o.), MOR (5 mg/kg, p.o.), or PECN (100, 250 or 500 mg/kg, p.o.) 60 min before the intraplantar (i.pl) administration of 5% (*v*/*v*) formalin into the right hind paw. The nociceptive response, indicated by the sum of time that the animal spent licking the injected paw, developed between 0-5 min after formalin injection (early phase) and 15–30 min after formalin injection (late phase) were recorded for 30 min.

### Determination of PECN effect on motor coordination

#### Rota–rod test

The method used was conducted similarly to the one that was described previously [11]. The apparatus consisted of a horizontal bar with a diameter of 3 cm, and subdivided into five compartments (UgoBasile, model 47600). The animals were placed on the rota–rod at a fixed speed of 20 revolutions per min. Those that were able to remain on the apparatus for 120 s without falling were selected 24 h before test. The mice (*n* = 6) were treated with the vehicle (10% DMSO, 10 mL/kg, p.o.), DZP (4 mg/kg, p.o., positive control), or PECN (500 mg/kg, p.o.) 60 min before the test. The average time the mice spent to stay on the rota–rod was measured for 120 s at 5, 10, and 15 min after the administration of PECN, respectively.

### Elucidation of the possible mechanisms of antinociception modulated by PECN

#### Effect of PECN on opioid-mediated nociception

The possible role of opioid receptor system in the modulation of PECN-induced antinociceptive activity was investigated as previously described [12]. Briefly, 10 mg/kg β–funaltrexamine (β–FNA; a μ-opioid antagonist), 1 mg/kg naltrindole (NALT; a δ-opioid antagonist) or 1 mg/kg nor–binaltorphimine (nor–BNI; a κ-opioid antagonist) were administered (i.p.) for the respective 90, 15 and 30 min before the administration of vehicle (10 mL/kg, p.o.) or PECN (500 mg/kg, p.o.). Sixty minutes after the administration of test solutions, the mice were subjected to acetic acid–induced abdominal writhing test as described above.

#### Effect of PECN on l–arginine/nitric oxide/cyclic guanosine monophosphate (l–arg/NO/cGMP) pathway-mediated nociception

To investigate the possible contribution of l–arg**/**NO**/**cGMP pathway to the antinociceptive effect of PECN, the previously described method was adopted from Jiménez–Andrade et al. [13]. The mice (*n* = 6) were pre–treated (i.p.) with 20 mg/kg l–arg (a NO precursor) or 2 mg/kg ODQ (a specific soluble guanylyl cyclase inhibitor) 5 min before the administration of vehicle (10 mL/kg, p.o.) or PECN (500 mg/kg, p.o.). Sixty minutes after the administration of test solutions, the mice were subjected to acetic acid–induced abdominal constriction test as described above.

### Phytoconstituents of PECN

#### Phytochemical screening of PECN

The qualitative phytochemical screening of PECN was performed to determine the presence of flavonoids, saponins, triterpenes, tannins, alkaloids and steroids according to the conventional protocols as adopted by Mamat et al. [14].

#### High performance liquid chromatography (HPLC) analysis of PECN

The HPLC analysis of PECN was conducted at the Laboratory of Phytomedicine, Forest Research Institute of Malaysia, Kepong, Malaysia according to the similar protocols as reported in Zakaria et al. [7]. The HPLC system consisting of Waters Delta 600 with 600 Controller equipped with Waters 996 photodiode array detector and a Phenomenex Luna column (5 μm; 4.6 mm i.d. × 250 mm) (Torrance, CA, USA) was used in this study. Briefly, the gradient elution program was as follows: initial conditions were 95% A (0.1% aqueous formic acid) and 5% B (acetonitrile) with a linear gradient reaching 25% B at t = 12 min. This condition was maintained for 10 min wherein at t = 22 min, B was reduced to 15% and then maintained until t = 30 min, after which the program returned to the initial solvent composition at t = 35 min. Chromatograms were recorded and integrated at peak areas on Millennium 32 Chromatography Software (Waters Co., Milford, MA). The peak elution was monitored at 210, 254, 280, 300, 330 and 366 nm. The retention times, peak areas and UV spectra of main peaks were examined. The HPLC profile of PECN was also compared with several known compounds, namely cinnamic acid, caffeic acid, coumaric acid, vanillic acid, ferulic acid, gallic acid, epicatechin gallate and catechin, at 254 nm. Retention times for the standard compounds and the major peaks in the extract were recorded. Identification of the possible bioactive compounds was made by comparing the similarity between the retention time and UV spectra information of detected peaks against those of the pure compounds.

### Statistical data analysis

Data are expressed as mean ± standard error of mean (SEM). Except for the hot plate test, the mean differences between the control and treatment groups for the other tests were analysed using the one–way analysis of variance (ANOVA) followed by Dunnet’s post–test (GraphPad Prism version 6.04; GraphPad Software, San Diego, CA, USA). Data for the hot plate test were analysed using the two-way ANOVA followed by the Bonferroni’s post hoc test. In all cases, the differences were considered as significant if *p* < 0.05.

## Results

### Effect of PECN on various models of nociception

**Fig. 1:**
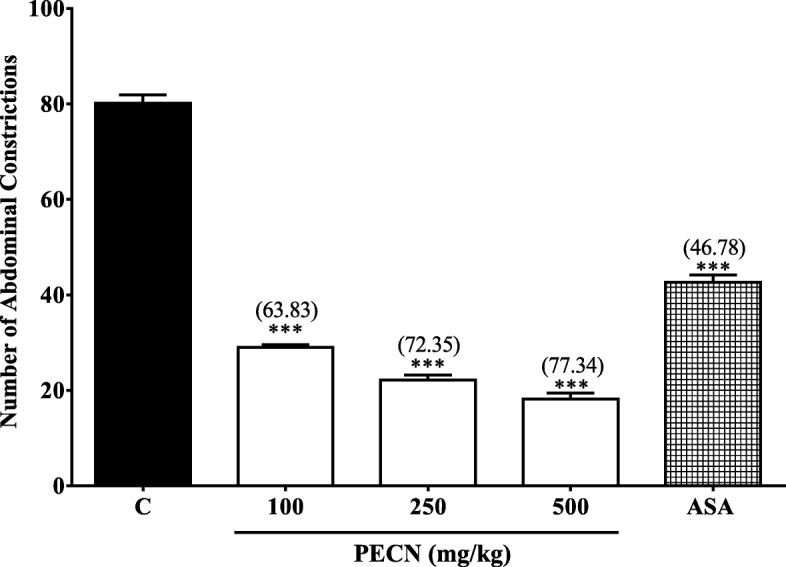
Antinociceptive effect of PECN assessed using the acetic acid–induced abdominal constriction in mice. Animals were treated with vehicle (10 mL/kg, p.o.), ASA (100 mg/kg, p.o.), or PECN (100, 250, 500 mg/kg, p.o.) 60 min before acetic acid (0.6%, 10 mL/kg, i.p.) treatment. Each column represents the mean ± SEM of six mice. Statistical analyses were performed using one–way ANOVA followed by Dunnett’s post hoc test. *** *p* < 0.001 compared to control group. Values in parentheses denote percentage of inhibition

The effect of PECN against acetic acid–induced abdominal constriction test in mice is shown in Fig. 1. PECN (100–500 mg/kg, p.o.) caused a significant (*p* < 0.001) and dose–dependent decrease in the number of abdominal constrictions in comparison to the control group. The percentage of antinociception calculated for the three respective doses of PECN ranged between 63.8 to 77.3%. ASA (100 mg/kg, p.o) also caused significant (*p* < 0.001) inhibition of abdominal constriction with the percentage of antinociception recorded at approximately 46.8%.

The effect of PECN on thermal-induced nociception was assessed using the hot plate test and is shown in Table 1. The extract, at all tested doses, caused significant (*p* < 0.01) changes in response latency to thermal stimulus–induced nociception, which started 60 min after PECN administration and lasted until the end of the experimentation when compared against the control group. Concurrently, MOR given via oral administration at the dose of 5 mg/kg also caused a highly significant (*p* < 0.001) prolongation of latency response starting at the interval of 60 min and lasted until the end of the experimentation (210 min).

**Table 1:** Antinociceptive effects of PECN assessed using the hot plate test in mice

Group	Dose (mg/kg)	Latency of discomfort(s) at respective time interval (min)						
		0 min	60 min	90 min	120 min	150 min	180 min	210 min
10% DMSO		6.29 ± 0.15	6.88 ± 0.29	6.89 ± 0.31	6.28 ± 0.12	6.76 ± 0.43	6.67 ± 0.33	6.46 ± 0.12
PECN	100	6.41 ± 0.40	8.95 ± 0.87^*a^	8.23 ± 0.35^*a^	8.38 ± 0.48^*a^	8.11 ± 0.66^*a^	8.41 ± 0.58^*a^	7.57 ± 0.63
	250	5.70 ± 0.16	9.71 ± 0.84^#b^	8.29 ± 0.40^#b^	8.92 ± 0.50^#ba^	8.17 ± 0.33^*b^	7.77 ± 0.31^*a^	7.89 ± 0.28^*a^
	500	6.37 ± 0.18	10.28 ± 0.72^§c^	9.52 ± 0.47^#b^	8.96 ± 0.23^#a^	9.47 ± 0.31^#b^	9.55 ± 0.40^#b^	9.06 ± 0.51^#b^
MOR	5	6.02 ± 0.15	17.00 ± 0.90^§c^	18.42 ± 0.47^§c^	17.25 ± 0.93^§c^	13.47 ± 1.31^§c^	11.87 ± 1.04^§c^	11.15 ± 0.71^§c^

Data expressed are the mean ± SEM of reaction time (sec) of six mice

Statistical analysis was performed using two–way ANOVA followed by the Bonferroni post hoc test

^*^p < 0.05, ^#^*p* < 0.01, ^§^*p* < 0.001, compared to control group within the respective column

^a^*p* < 0.05, ^b^*p* < 0.01, ^c^*p* < 0.001, compared to control group within the respective row

*DMSO* Dimetyl sulfoxide, *PECN* Petroleum ether extract of *C. nutans*, *MOR* morphine

Mice were treated (p.o) with vehicle (10 mL/kg), PECN (100, 250, 500 mg/kg), or MOR (5 mg/kg) for 60 mins prior to subjection to the test

**Fig. 2:**
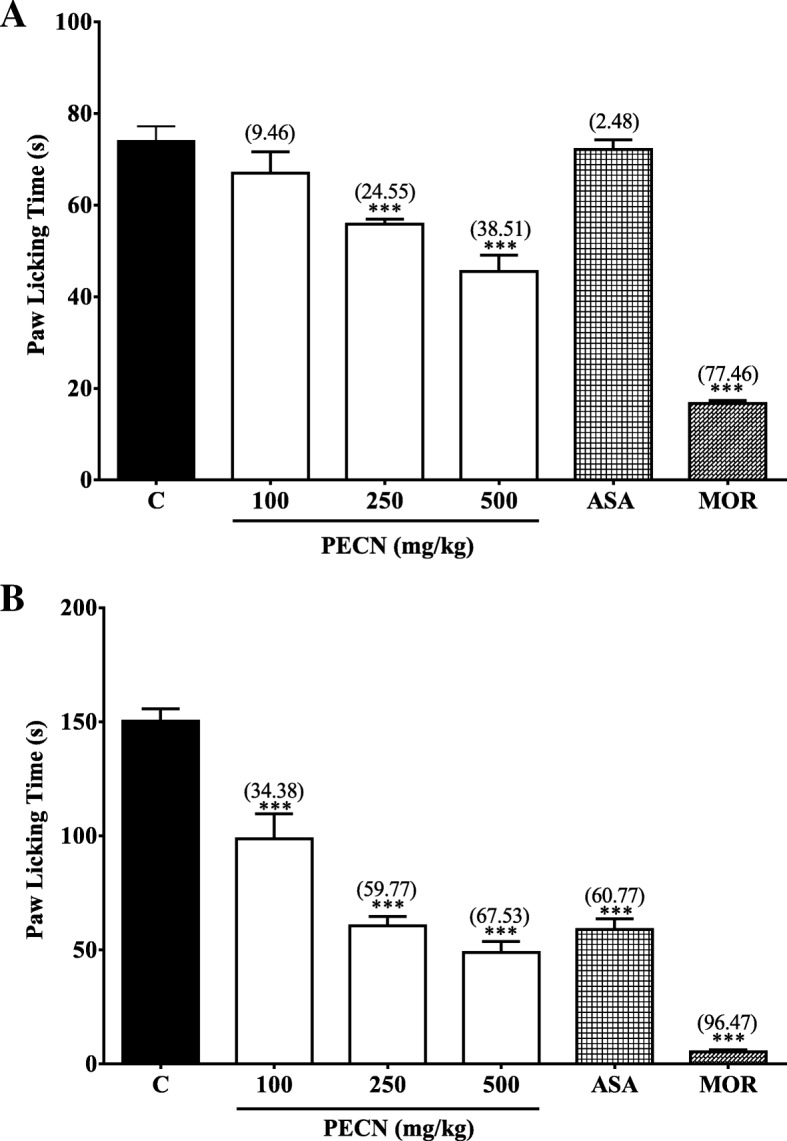
Antinocicpetive effect of PECN assessed using the formalin–induced paw licking in mice (**a**) Early phase; (**b**) late phase. Animals were treated with vehicle (10 mL/kg, po), ASA (100 mg/kg, po), PECN (100, 250, 500 mg/kg, po), or MOR (5 mg/kg, po) 60 min before intraplantar administration of formalin (5% in normal saline; 20 μL) into the right hind paw. Each column represents the mean ± SEM of six mice. Statistical analyses were performed using one–way ANOVA followed by Dunnett’s post hoc test. ^***^p < 0.001 compared to control group. Values in parentheses denote percentage of inhibition

Figure 2 shows the effect of PECN against formalin–induced paw licking test. The extract, at the doses of 250 and 500 mg/kg, caused significant (*p* < 0.001) reduction in the latency of formalin–induced paw licking in the first phase (neurogenic phase; 0–5 min) of the test as shown in Fig. 2a. The percentage of antinociception recorded for the three doses of PECN ranged between approximately 9.5 to 38.5% in comparison to ASA (≈2.5%) and MOR (≈77.5%). It is noticeable that the centrally- but not peripherally-acting analgesic was more effective in relieving neurogenic pain. Moreover, the extract also significantly (*p* < 0.001) reduced the latency of formalin–induced paw licking in the second phase (inflammatory phase; 15–30 min) of the test as shown in Fig. 2b. The range of percentage of antinociception recorded for the three doses of PECN was between 34.4 to 67.5% in comparison to ASA (60.8%) and MOR (96.5%). It is noticeable that the centrally- as well as peripherally-acting analgesics were effective in relieving inflammatory-mediated pain.

**Fig. 3:**
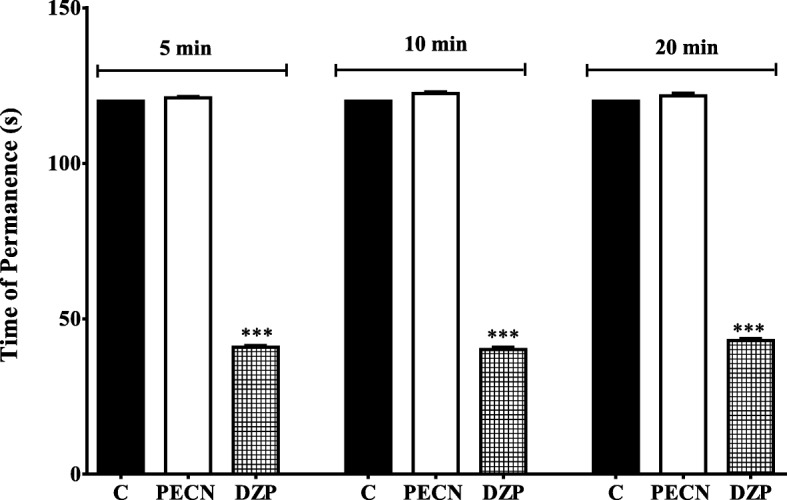
Effect of PECN on motor co-ordination assessed using the rota–rod test in mice. Animals were treated with vehicle (10 mL/kg, p.o.), DZP (4 mg/kg, p.o.), PECN (500 mg/kg, p.o.) 60 min before the rota–rod test. Each column represents the mean ± SEM of six mice. Statistical analyses were performed using one–way ANOVA followed by Dunnett’s post hoc test. *** *p* < 0.001 compared to control group

The effect of orally administered PECN on motor coordination of mice was measured using the rota–rod test and is shown in Fig. 3. PECN, at the dose of 500 mg/kg, did not cause any significant change on the latency as the mice remained on the rota rod at all interval (5, 10 and 20 min) when compared to the control group. On the other hand, 4 mg/kg DZP administered orally caused significant (*p* < 0.001) reduction in the latency spend by the mice to remain on the rota-rod at all interval measured indicating its potential to affect motor coordination.

### Possible mechanisms of antinociception modulated by PECN

#### Involvement of opioid receptor system

**Fig. 4:**
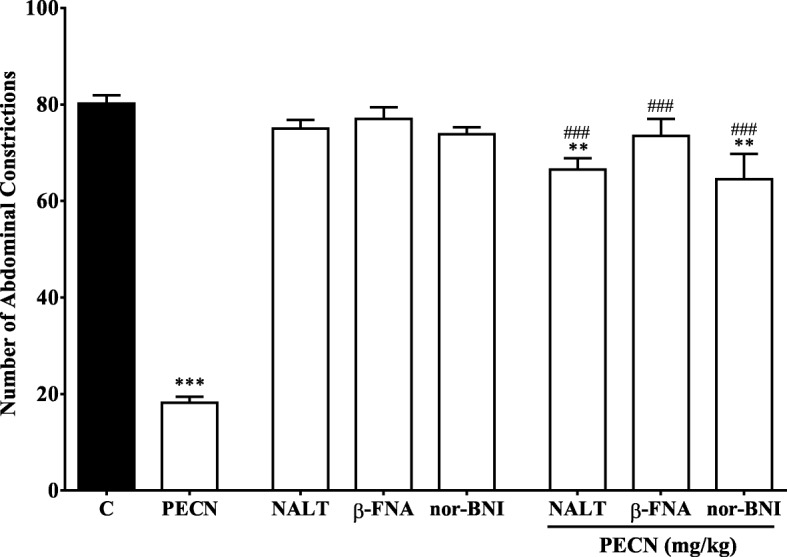
Effect of opioid receptor antagonists on PECN–induced antinociception assessed using the acetic acid–induced abdominal constriction test in mice. *β*–funaltrexamine (β–FNA; 10 mg/kg, i.p.), naltrindole (NALT; 1 mg/kg, i.p.), or nor–binaltorphimine (nor–BNI; 1 mg/kg, i.p.) were administered 90 min, 15 min and 30 min respectively, before oral administration of vehicle (10 mL/kg), or PECN (500 mg/kg). Each column represents the mean ± SEM of six mice. Statistical analyses were performed using one–way ANOVA followed by Dunnett’s post hoc test. ^**^ *p* < 0.01, ^***^ *p* < 0.01compared to control group. ^###^ *p* < 0.001 compared to 500 mg/kg PECN–treated group

Effect of PECN on opioid receptors-mediated nociceptive response was determined using the acetic acid-induced abdominal constriction test and is shown in Fig. 4. The antinociceptive activity of PECN, at the dose of 500 mg/kg, was significantly (*p* < 0.001) reversed after i.p. pretreatment with 10 mg/kg β–FNA, 1 mg/kg NALT or 1 mg/kg nor–BNI, which did not exert any significant (*p* > 0.05) effect on nociceptive response when tested alone.

#### Involvement of l–arg/NO/cGMP pathway

**Fig. 5:**
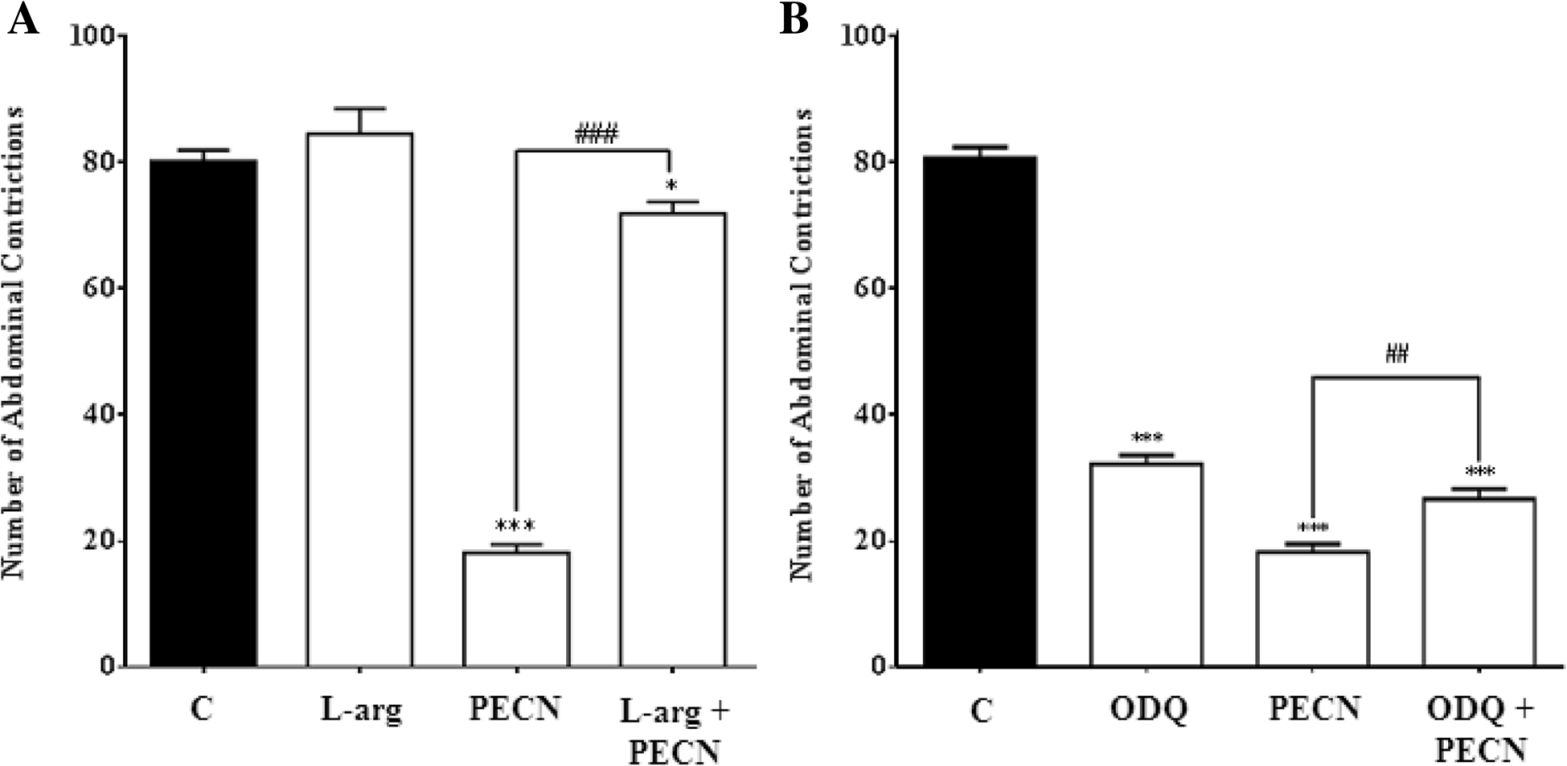
Effects of l–arg (**a**) or ODQ (**b**) on PECN-induced antinocicpetive activity assessed using the acetic acid–induced abdominal constriction test in mice. Animals were treated with PECN (500 mg/kg, p.o.) or vehicle (10 mL/kg, p.o.) 60 min before acetic acid (0.6%, 10 mL/kg, i.p.) treatment. l–arg (20 mg/kg, i.p.) or ODQ (2 mg/kg, i.p.) were administered 5 min before PECN (500 mg/kg, p.o.) or vehicle (10 mL/kg, p.o.). Each column represents the mean ± SEM of six mice. Statistical analyses were performed using one–way ANOVA followed by Dunnett’s post hoc test. *p < 0.05, ***p < 0.001 compared to control group; ^#^*p* < 0.05, ^##^*p* < 0.01, and ^###^*p* < 0.001 compared to 500 mg/kg PECN–treated group

The involvement of l–arg**/**NO**/**cGMP pathway in the modulation of PECN-induced antinociception is shown in Fig. 5. Effects of l–arg or ODQ, alone or in combination with PECN, were assessed using the acetic acid–induced abdominal constriction test. As shown in Fig. 5a, l–arg (the NO precursor) alone did not affect the acetic acid-induced nociception but caused significant reversal (*p* < 0.001) on PECN-induced antinociception when pretreated together. Moreover, ODQ, given alone, caused significant (*p* < 0.001) attenuation of the acetic acid–induced nociception while pretreatment with PECN caused significant (*p* < 0.01) reversal of the antinociceptive activity of PECN (Fig. 5b).

### Phytoconstituents of PECN

#### Phytochemical screening

The qualitative analysis of PECN revealed the presence of flavonoids, saponins, steroids and triterpenes, but the absence of alkaloids and tannins.

### HPLC analysis

**Fig. 6:**
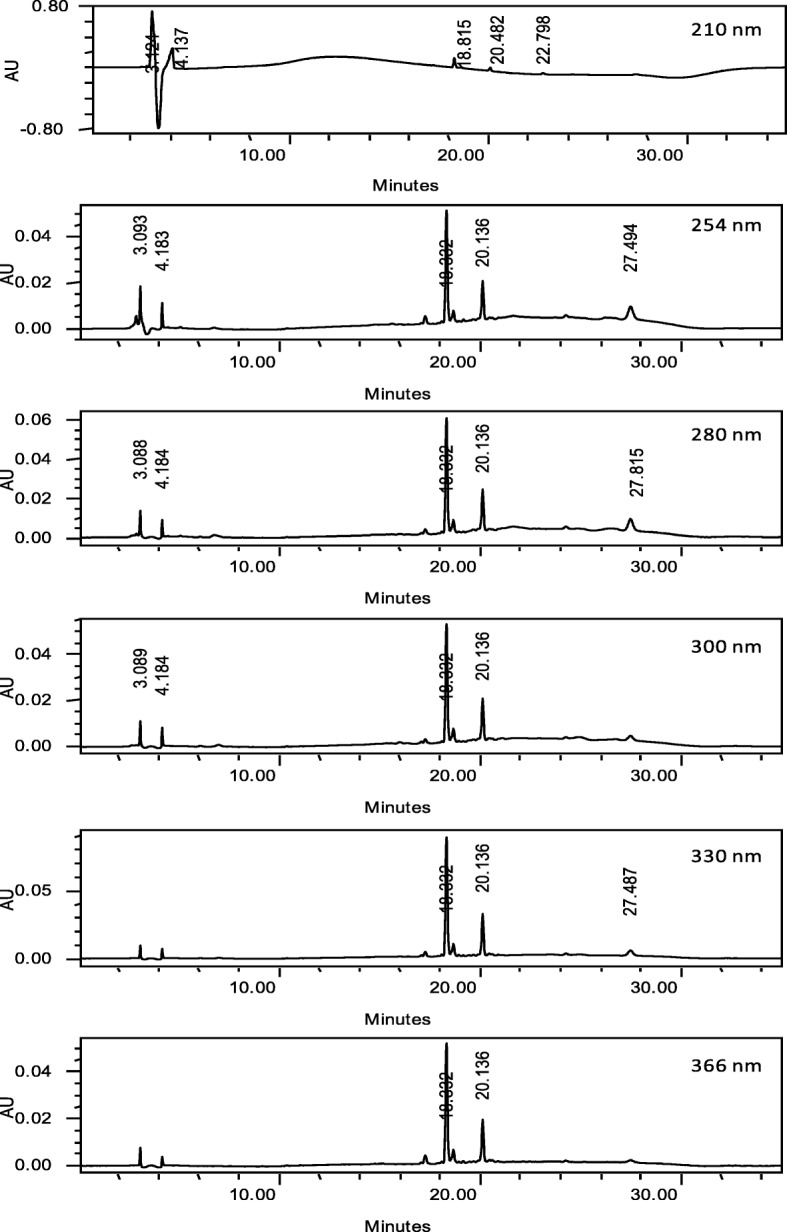
The HPLC profile of PECN at the wavelengths of 210, 254, 280, 300, 330 and 366 nm

**Fig. 7:**
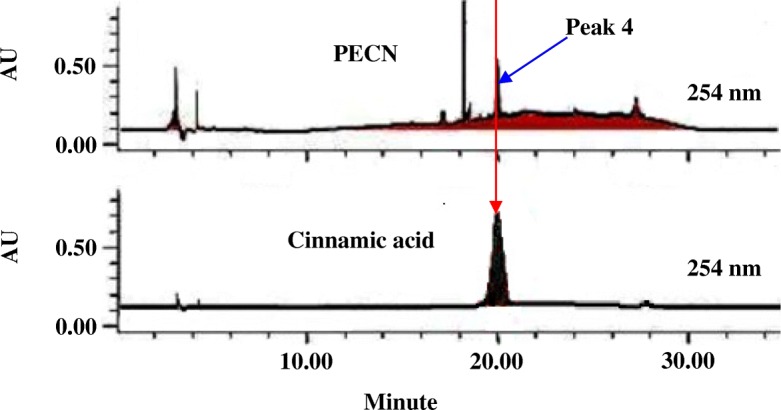
Comparison of chromatogram between PECN and several phenolic compounds at the wavelength of 254 nm demonstrated one peak that was believed to represent (Peak 4) cinnamic acid. Only chromatogram of cinnamic acid was presented for comparison purposes

The HPLC profiles of PECN were measured at six different wavelengths, namely 210, 254, 280, 300, 330 and 366 nm to obtain the best separation. Based on the respective chromatograms obtained, chromatogram established at the wavelength of 254 nm was found to show the best peaks separation (Fig. 6). The UV spectra analysis of PECN demonstrated the presence of five major peaks labelled as P1 (retention time (R_T_) = 17.265 min), P2 (retention time (R_T_) = 18.332 min), P3 (R_T_ = 18.631 min), P4 (R_T_ = 20.136 min) and P5 (R_T_ = 27.494 min). Further analysis demonstrated that the three major peaks showed their λ_max_ values in the region of 222.5–332.6, 192.0–336.2, and 216.6–335.0 nm, respectively, thus, suggesting the presence of phenyl chroman (C_6_–C_3_–C_6_) skeletal structures. As depicted in Fig. 7, a comparison between chromatogram of the standard compounds with chromatogram of PECN revealed the presence of cinnamic acid.

## Discussion

The current work is a continuation of previous study by Abdul Rahim et al. [6] on the antinociceptive activity and possible mechanisms of action of MECN. In an attempt to isolate the bioactive compound(s) with antinociceptive potential from *C. nutans*, MECN was sequentially partitioned based on their polarity to obtain the petroleum ether (PECN), ethyl acetate and aqueous semi-purified fractions [8], which were later assessed for their potential to attenuate nociceptive response using the acetic acid-induced abdominal constriction test. From the results obtained, PECN was found to exert the most effective antinociceptive activity (data not shown) and, thus, subjected to further antinociceptive study.

Other than the acetic-acid-induced abdominal constriction test, PECN was found to attenuate nociceptive response when assessed using the hot plate test and formalin-induced paw licking test. The acetic acid–induced abdominal constriction test, which represents inflammatory–mediated peripheral nociceptive response, has been associated with increased production of prostaglandin (PG) (such as PGE_2_ and PGF_2_), lipoxygenase (LOX) and cyclooxygenase (COX) in the peritoneal cavity that lead to indirect liberation/activation of various endogenous inflammatory mediators. These mediators stimulate the peripheral nociceptive neurons that are sensitive to opioid (i.e. MOR) and non-opioid (i.e. ASA) analgesics [15]. The ability of PECN to inhibit acetic acid–induced nociception indicated that the extract possesses a peripherally-mediated antinociceptive activity possibly via the inhibition of inflammatory mediators action, attenuation of the PG level, and reduction of LOX and COX production/activities. Although considered as a dose/concentration–sensitive nociceptive model, the abdominal constriction test has been acknowledged to have a poor specificity since non-analgesic drugs such as muscle relaxants can also produce false positive results [10]. Due to this issue, the abdominal constriction test could not be solely used to stipulate the involvement of peripheral and/or central antinociceptive mechanisms of a compound/drug, including PECN [16]. Therefore, additional experimentations using other nociceptive assays are necessary to ensure that the correct conclusion could be made on the antinociceptive mechanisms of PECN, as well as, other pain relieving agents.

Therefore, the antinociceptive potential of PECN was further investigated using the hot plate test in mice. This thermal-induced nociceptive assay, considered to represent a centrally-mediated nociceptive model as it is sensitive only to the centrally-acting analgesic drug (i.e. MOR) [17], involves a supraspinally-integrated nociceptive responses [18]. According to Imam and Sumi [19], centrally-acting analgesic drugs activate the liberation of endogenous peptide through the periaqueductal gray matter consequently leading to inhibition of nociceptive impulse transmission within the dorsal horn. Interestingly, PECN was also able to inhibit nociceptive response assessed using the hot plate test signifying its centrally-mediated pain relieving potential. However, the intensity of pain relief exerted by MOR was greater than all doses of PECN tested.

Based on its ability to attenuate both the acetic acid- and thermal-induced nociceptive responses, PECN is suggested to have a potential to inhibit nociceptive response at the peripheral and central levels [20–22]. This suggestion is based on previous reports that demonstrated ASA (a peripherally-acting analgesic) ability to attenuate only the acetic acid-induced nocieptive response [20] and MOR (a centrally-acting analgesic) potentials to inhibit both models of nociception [21, 23]. Interestingly, the ability of PECN to exert peripherally and centrally-mediated antinociception was further confirmed by the extract ability to attenuate both the early and late phases of formalin-induced paw licking test. This suggestion was made based on previous reports that ASA only inhibits the late phase whereas MOR inhibits both phases of the formalin-induced test [7, 8, 12]. It is also worth-mentioning that the ability of PECN to reduce nociception in the early and late phases indirectly implied its potential to attenuate non-inflammatory- and inflammatory-mediated nociceptive responses, respectively.

Although the three nociceptive assays used in the present study finally established that PECN possesses antinociceptive potential, it is necessary to determine the extract ability to modulate motor coordination of the mice upon consumption due to its centrally-acting effect since centrally-acting drugs are known to cause possible alterations in the motor coordination, balance and equilibrium ability of the animals [24]. According to Rabelo et al. [25], centrally-acting drugs can depress the CNS or trigger the non-specific muscle relaxation leading to decrease in the motor coordination response. Consequently, this may lead to misinterpretation of the nociceptive behavioural results. Based on the results obtained, the centrally-acting PECN did not interfere with the motor coordination of mice when assessed using the rota-rod test thereby eliminates the non-specific effects such as muscle relaxation and sedation [26]. Based on this finding, it is safe to suggest that the bioactive compounds present in PECN triggered the observed antinociceptive activity particularly at the central level without affecting the mice motor coordination.

In an attempt to establish the antinociceptive profile of PECN, the mechanisms of antinociception modulated by PECN were also investigated by focusing on the role of opioid receptor systems (such as μ-, δ- and κ-receptor subtypes) and l–arg/NO/cGMP pathway. The opportunity to study and identify any drugs/substances that modulate nociceptive response via mechanisms partly used by standard analgesic (MOR) (viz. opioid receptors and l–arg**/**NO**/**cGMP pathway) could be an added advantage in an attempt to find replacement to MOR, which has been associated with side effects such as dependence and tolerance.

Role of opioid receptor system in the modulation of antinociceptive action of drugs/substances can be determined via exploitation of certain pharmacological tools such as various antagonists of opioid receptor [27, 28]. The opioidergic receptor system involvements in the transmission and modulation of nociceptive response have been greatly acknowledged [29]. The most common and well-known opioid analgesic is MOR, which has been considered as the gold standard analgesic drug. However, the effectiveness of MOR and other opioids has been surpassed by the adverse effects associated with their prolonged use [30]. Thus, there is a need to look for an alternative or new analgesic candidate drug(s) that can replace MOR in the future. Concurrent with this need, the role of opioid receptors’ subtypes, namely ü-, κ- and δ-subtypes, in the modulation of PECN-induced antinociceptive activity was measured using the abdominal constriction test. In the present study, all antagonists of opioid receptor subtypes, namely β–FNA (a μ-opioid antagonist), nor–BNI (a κ-opioid antagonist) or NALT (a δ-opioid antagonist), caused complete inhibition of PECN-induced antinociceptive activity. This finding was paralleled with previous report on the ability of these opioid antagonists to completely attenuate MECN-induced antinociceptive activity [7], which seems to suggest that PECN also possesses a non-selective opioid activity. In line with this suggestion, several drugs like bremazocine and buprenorphine have also been reported to demonstrate a non-selective opioid-mediated antinociceptive activity [31, 32].

The involvement of l–arg/NO/cGMP pathway in the regulation of analgesics action at peripheral and central levels haa been reported [33]. NO, derived from the conversion of l–arg to l–citrulline and NO by NO synthase, is known to play a complex part in the transmission of nociceptive signals peripherally and centrally [34]. In fact, depending on the nociceptive system level (central or peripheral) and the amount of NO, it could have either antinociceptive or pronociceptive actions. In addition, NO also demonstrated dual actions (hyper- and anti-nociception) even at the periphery sites (epidermis vs. dermis) suggesting that each tissue might be primarily innervated by diverse subsets of primary nociceptive neurons [35]. Although l–arg exerted insignificant increase in the nociceptive intensity when administered alone, it significantly reduced the antinociceptive intensity of PECN when pretreated together indicating the importance of NO presence in the modulation of PECN antinociceptive potential. On the other hand, increased presence of NO at the intracellular level stimulates the soluble guanylate cyclase (sGC), which augments the cGMP synthesis. The important of cGMP in the modulation of hyperalgesic response at peripheral sensory neurons and the involvement of NO/cGMP signaling pathway in the central and peripheral nociceptive processing have been established [36]. In the present study, ODQ, an inhibitor of sGC, exerted antinociceptive activity when given alone but failed to significantly alter PECN antinociceptive activity, thus, suggesting the involvement of cGMP-independent pathway in the observed antinociceptive activity. Interestingly, this finding was in agreement with our earlier report on the involvement of NO/cGMP-independent pathway in the modulation of antinociceptive activity of MECN [6]. One example of cGMP-independent mechanism of NO signaling is S-nitrosylation, which refers to the covalent and reversible attachment of NO to a reactive cysteine thiol. Various studies have indicated that S-nitrosylation is a signaling mechanism of NO during pain processing [37, 38]. Taking into consideration that the l–arg/NO/cGMP pathway plays a pivotal role in the development of tolerance to MOR [39], it is reasonable to develop *C. nutans* as an analgesic-based product with lack of unwanted side effects seen with MOR due to the difference in NO-mediated pathway modulated by the former.

Earlier investigation on the phytochemical content of *C. nutans* leaves demonstrated the presence of flavonoids, phenolic compounds, diterpenes, saponins, chlorophyll and its various derivatives, and steroids (i.e. *β*–sitosterol and stigmasterol) [4, 5]. Concurrently, the qualitative phytochemical analysis of PECN also showed the presence of flavonoids, saponins, steroids and triterpenes. Interestingly, various reports have shown that flavonoids [40, 41], triterpenes [42, 43], and saponins [44, 45] exert antinociceptive activity, which might explain the ability of PECN to induce antinociceptive activity.

Further phytoconstituents analysis using HPLC revealed the presence of traces of only a few bioactive compounds (indicated by the presence of small numbers of small-sized peaks), which upon comparison with several pure bioactive compounds revealed the presence of, at least, cinnamic acid. The lack of number of peaks and the presence of small peaks as detected in the chromatogram of PECN might be due to the lack of hydrophilic (water-soluble) bioactive compounds in the hydrophobic-based extract. The presence of cinnamic acid in PECN is parallel to the report of Tüzan and Özdemir [46] on the presence of this acid in petroleum ether extract of *Galanthus elwesii*. Concurrent with this finding, there are reports on the potential of cinnamic acid derivatives [47] to exert antinociceptive activity.

## Conclusion

In conclusion, PECN, partly through the presence of cinnamic acid, exerted antinociceptive activity at the peripheral and central levels via the non-selective activation of opioid receptors and modulation of the l–arg/NO-mediated, but cGMP-independent, pathway.

## Abbreviations

ASA: Acetylsalicylic acid
*C. nutans*: *Clinacanthus nutans*
cGMP: Cyclic guanosine monophosphate
CNS: Central nervous system
COX: Cyclooxygenase
DMSO: Dimethyl sulfoxide
DZP: Diazepam
IACUC: Institutional Animal Care and Use Committee
IBS: Institute of Bioscience
l-arg: l-arginine
l–arg: l–arginine
LOX: Lipoxygenase
MECN: Methanolic extract of *C. nutans*
MeOH: Methanol
MOR: Morphine hydrochloride
NALT: Naltrindole hydrochloride
NO: Nitric oxide
nor–BNI: Nor–binaltorphimine dihydrochloride
NSAIDs: Nonsteroidal anti-inflammatory drugs
ODQ: 1H-[1, 2, 4] oxadiazole [4,3–a]quinoxaline–1–one
PE: Petroleum ether
PECN: Petroleum ether fraction of MECN
PG: Prostaglandin
PGE_2_: Prostaglandin E_2_
PGF_2_: Prostaglandin F_2_
PNS: Peripheral nervous system
R_T_: Retention time
SEM: Standard error of mean
sGC: Soluble guanylate cyclase
UPM: Universiti Putra Malaysia
UV: Ultra violet
β-FNA: β–funaltrexamine hydrochloride
